# Anti-inflammatory effect of thalidomide in an experimental lung donor model of brain death

**DOI:** 10.1038/s41598-024-59267-1

**Published:** 2024-04-16

**Authors:** Vanessa Sana Vilela, Karina Andrighetti de Oliveira Braga, Liliane Moreira Ruiz, Natalia Aparecida Nepomuceno, Paolo Oliveira Melo, Giovana Maria Manzuti, Vinícius Alcantara de Oliveira Costa, Jhonatan de Campos Ramos, Aristides Tadeu Correia, Paulo Manuel Pêgo-Fernandes

**Affiliations:** 1 Laboratorio de Pesquisa em Cirurgia Toracica, Instituto do Coração, Hospital das Clinicas HCFMUSP, Faculdade de Medicina, Universidade de Sao Paulo, Sao Paulo, SP Brazil; 2 Departamento de Cardiopneumologia, Laboratorio de Pesquisa em Cirurgia Toracica, Instituto do Coração, Hospital das Clinicas HCFMUSP, Faculdade de Medicina, Universidade de Sao Paulo, Sao Paulo, SP Brazil; 3 Laboratorio de Pesquisa em Cirurgia Toracica, Instituto do Coração, Hospital das Clinicas HCFMUSP, Faculdade de Medicina, Universidade de Sao Paulo, Rua Dr. Eneas de Carvalho Aguiar 44, bloco 1, SS, sala 25, Cerqueira Cezar, Sao Paulo, SP 05403-000 Brazil

**Keywords:** Medical research, Experimental models of disease, Translational research, Respiratory tract diseases

## Abstract

Lung transplantation stands as a vital treatment for severe lung diseases, primarily sourcing organs from donors with brain death (BD). This research delved into the potential anti-inflammatory effects of thalidomide in rats with BD-induced lung complications. In this study twenty-four Wistar rats were divided into three groups: the control (CTR), brain death (BD) and brain death + thalidomide (TLD) groups. Post specific procedures, a 360 min monitoring period ensued. Comprehensive analyses of blood and heart-lung samples were conducted. Elevated IL-6 levels characterized both BD and TLD groups relative to the CTR (p = 0.0067 and p = 0.0137). Furthermore, TNF-α levels were notably higher in the BD group than both CTR and TLD (p = 0.0152 and p = 0.0495). Additionally, IL-1β concentrations were significantly pronounced in both BD and TLD compared to CTR, with the BD group surpassing TLD (p = 0.0256). Immunohistochemical assessments revealed augmented NF-ĸB expression in the BD group in comparison to both CTR and TLD (p = 0.0006 and p = 0.0005). With this study we can conclude that BD induced acute pulmonary inflammation, whereas thalidomide manifested a notable capability in diminishing key inflammatory markers, indicating its prospective therapeutic significance in lung transplantation scenarios.

## Introduction

Lung transplantation is the main treatment option for patients with end-stage chronic lung disease. However, despite advances in the medical field, there is still a shortage of lungs suitable for donation. Organ harvesting occurs mainly from donors diagnosed with brain death (BD). However, the BD process triggers haemodynamic and hormonal cascades and an intense inflammatory process that can impair the function and quality of the lungs, impacting the survival of the transplant patient^1–4^.

BD begins with increased intracranial pressure, leading to total and irreversible loss of brain functions. The lung is one of several organs most vulnerable to the deleterious effects of BD since the increased production of proinflammatory factors favours the induction of acute lung injury and, therefore, may increase the incidence of primary graft dysfunction^3–7^.

Among the various immunological changes that occur during BD is the activation of the transcription factor nuclear factor kappa B (NF-κB), notable for contributing to the progression of transplant rejection through the expression of important genes with inflammatory activity. Experimental studies have shown that during BD, cytokines such as interleukin-1 beta (IL-1β), interleukin-6 (IL-6), and tumour necrosis factor alpha (TNF-α) are systemically released. The increased expression of these cytokines in the donor may contribute to the progressive deterioration of graft function associated with vascular and interstitial morphological changes during organ reperfusion. This donor condition is related to the primary dysfunction of the lung graft after transplantation^8,9^.

Thalidomide is a synthetic agent derived from glutamic acid that could serve as a potential interventional drug for controlling and/or reducing inflammation because it has important anti-inflammatory and immunomodulatory effects; consequently, it has been effectively used for the treatment of patients with inflammatory disorders since it acts by suppressing NF-κB while stimulating the production of anti-inflammatory cytokines such as interleukin-10 (IL-10)^10–12^.

Considering the importance of donor care and the anti-inflammatory and immunomodulatory effects of thalidomide, we analysed its effect in the treatment of lung inflammation in an experimental BD donor model.

## Results

### Haemodynamic parameters

**Figure 1:**
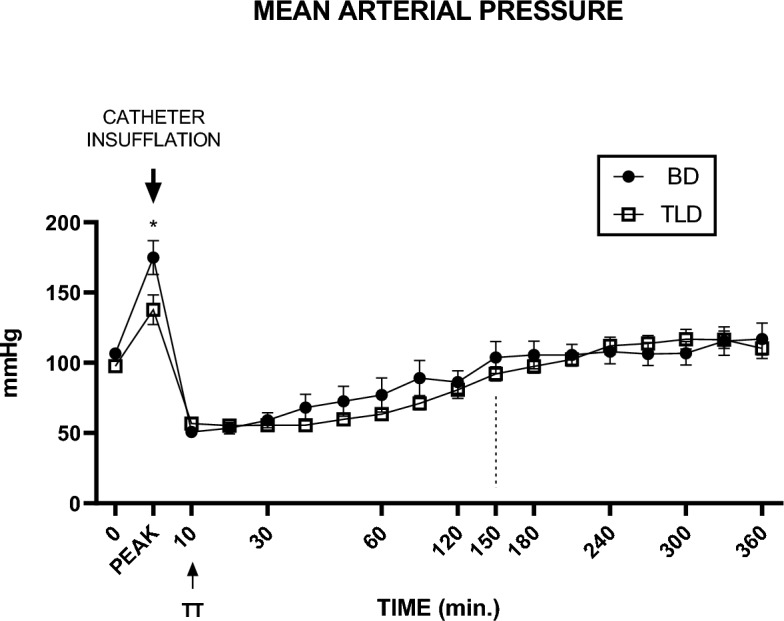
MAP monitoring in the TLD and BD groups during the 360 min of the experiment. Values are expressed as the mean ± standard error of the mean, and a p value < 0.05 was considered to indicate statistical significance. Two-way ANOVA followed by Sidak’s post hoc test. TTO, treatment. *p = 0.0098.

There were no differences in the baseline MAP between the BD and TLD groups. In the BD groups (BD and TLD), there was a significant increase in the MAP immediately after catheter insufflation, followed by arterial hypotension. 10 min after BD induction, both groups received treatment intraperitoneally. The MAP returned to baseline levels after 150 min (Fig. 1).

### Wet/dry weight ratio

**Figure 2:**
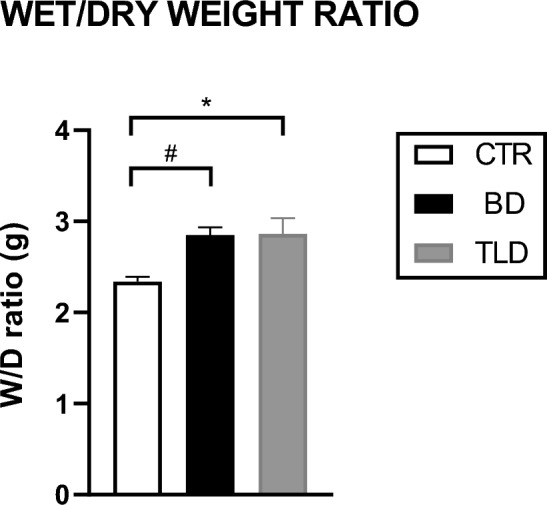
Analysis of the wet/dry weight ratio Values are expressed as the mean ± standard error of the mean, and groups were tested using analysis of variance (one-way ANOVA) with Tukey’s post hoc test. A p value of < 0.05 was considered to indicate statistical significance. ^#^p = 0.0083; *p = 0.0092.

In the analysis of the wet/dry weight ratio, the animals in the BD and TLD groups were significantly 1.2 times higher than the CTR group (Fig. 2).

### Inflammatory mediators and oxidative stress

The expression of marker IL-10 did not significantly differ between the groups (p = 0,7154) (data not shown).

**Figure 3:**
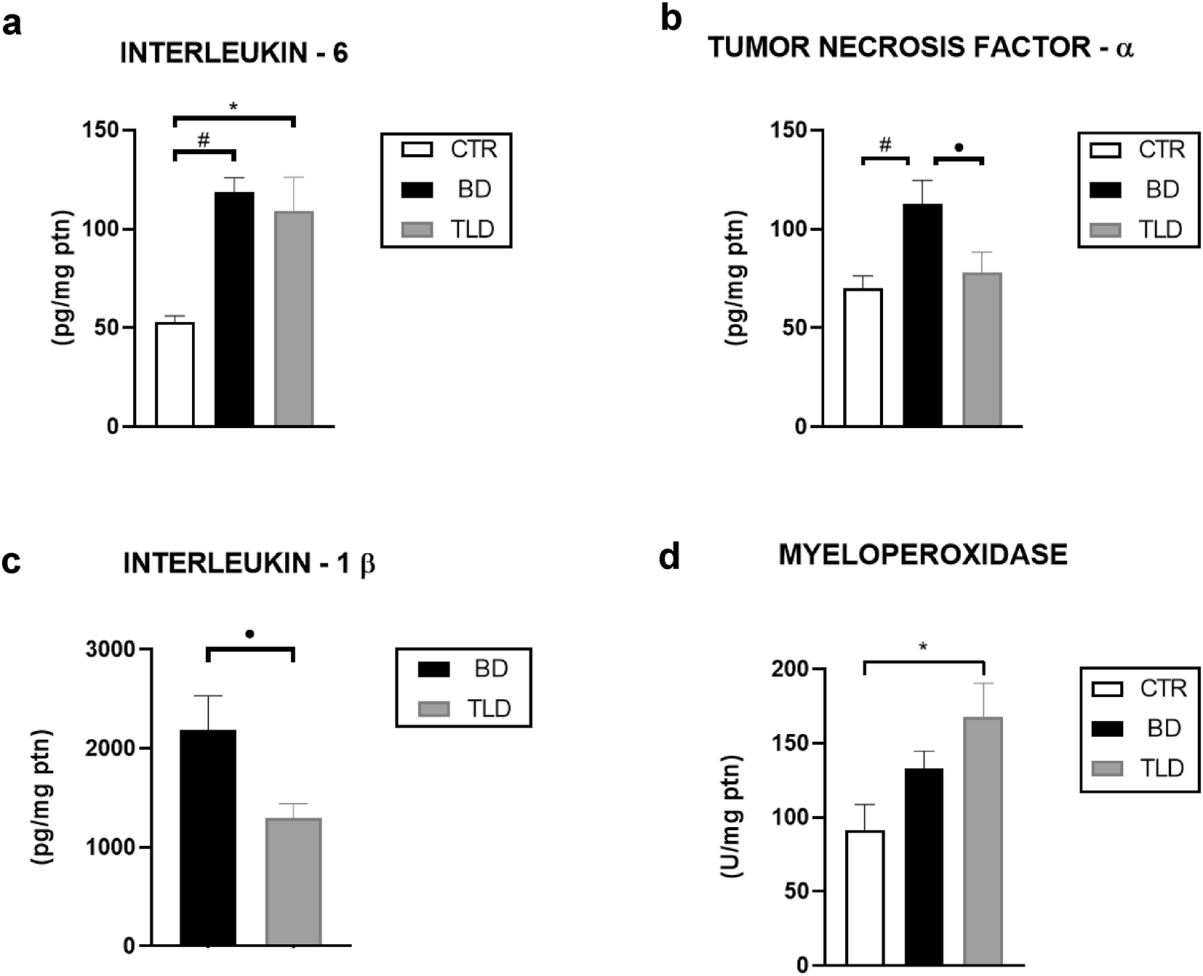
Analysis of IL-6 (**a**), TNF-α (**b**), and IL-1β (**c**) levels and myeloperoxidase activity (**d**) in lung homogenates. Values are expressed as the mean ± standard error of the mean, and groups were tested using analysis of variance (one-way ANOVA) with Tukey’s post hoc test (Fig. 3a,b,d) and Student’s t test (Fig. 3c**)**. A p value of < 0.05 was considered to indicate statistical significance.

We observed that, compared with that in the CTR group, IL-6 production in the BD and TLD groups was increased 2.2 and 2 times more respectively (Fig. 3a).

In the measurement of TNF-α levels showed that the values in the BD group were higher than those of the CTR and TLD groups. However, when comparing the CTR group with the TLD group, we observed that the TLD group-maintained TNF production close to the CTR values (Fig. 3b).

The levels of IL-1β were not detected in the control group but were higher in the BD group 1.6 times more than in the TLD group (Fig. 3c).

The neutrophilic infiltrate was evaluated by the activity level of, an enzyme released by neutrophils. MPO activity was 1.8 times more elevated in the TLD group compared to that in the CTR group (Fig. 3d).

**Figure 4:**
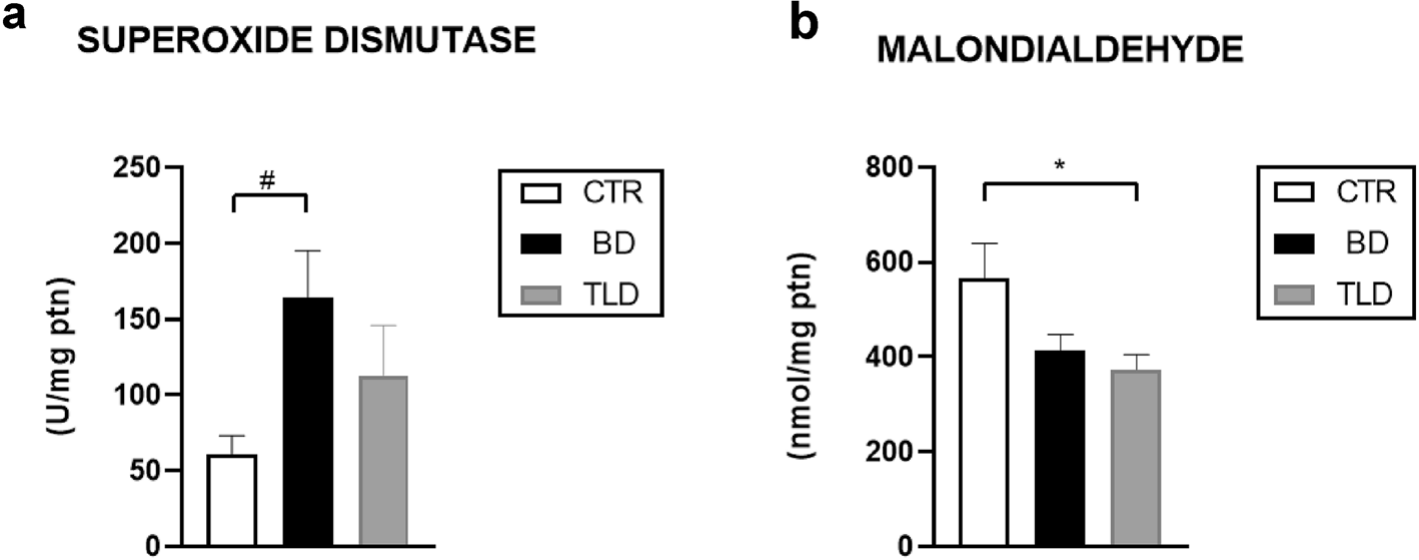
Analysis of superoxide dismutase (**a**) and malondialdehyde (**b**) levels in lung homogenates. Values are expressed as the mean ± standard error of the mean, and groups were tested using analysis of variance (one-way ANOVA) with Tukey’s post hoc test. A p value of < 0.05 was considered to indicate statistical significance.

SOD activity in the pulmonary homogenates was significantly 2.7 times higher in the BD group than in the CTR group. We found no significant difference between the TLD group and the CTR or BD group (Fig. 4a).

In our model, lipid peroxidation was analyzed via the levels of MDA in pulmonary homogenates. We observed 1.5 times more MDA levels in the CTR group than in the TLD group. No significant differences were observed between the BD and TLD groups, however (Fig. 4b).

The catalase and ROS levels were similar in the studied groups; no significant differences were observed (data not shown).

### Pulmonary immunohistochemistry

**Figure 5:**
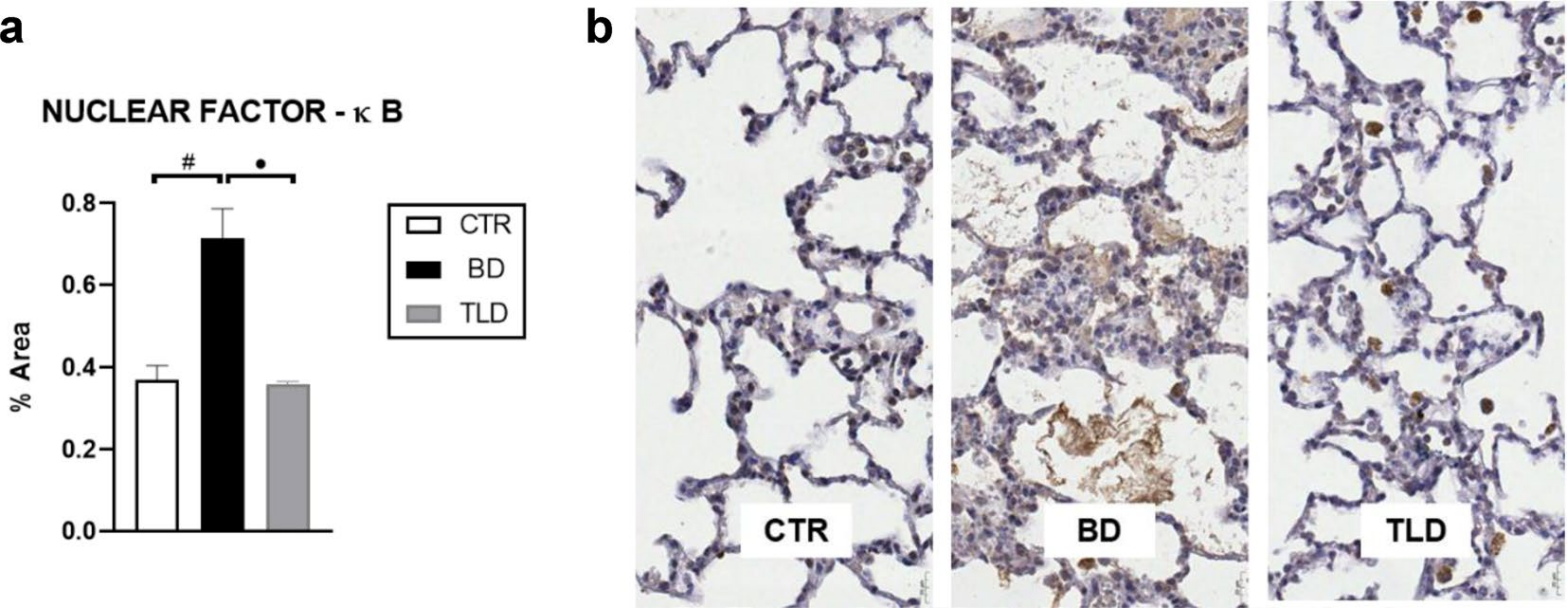
Evaluation of the level of the marker NF-κB in a lung tissue sample (**a**) and immunoexpression of the anti-NF-κB (p65) antibody (**b**). Values are expressed as the mean ± standard error of the mean, and groups were tested using analysis of variance (one-way ANOVA) with Tukey’s post hoc test. A p value of < 0.05 was considered to indicate statistical significance.

According to the immunohistochemical analysis performed by staining lung tissue slides with NF-ĸB antibody (p65), the level of NF-ĸB was increased by 1.9 times in the BD group compared to the CTR group and the TLD group. There were no statistically significant differences between the CTR and TLD groups (Fig. 5).

### Pulmonary histopathology

In the analysis of the area of perivascular oedema, no significant differences were observed among the groups (p = 0.1464) (data not shown).

Similar results were observed in the analysis of the lung injury score (p = 0.5078) (data not shown).

**Figure 6:**
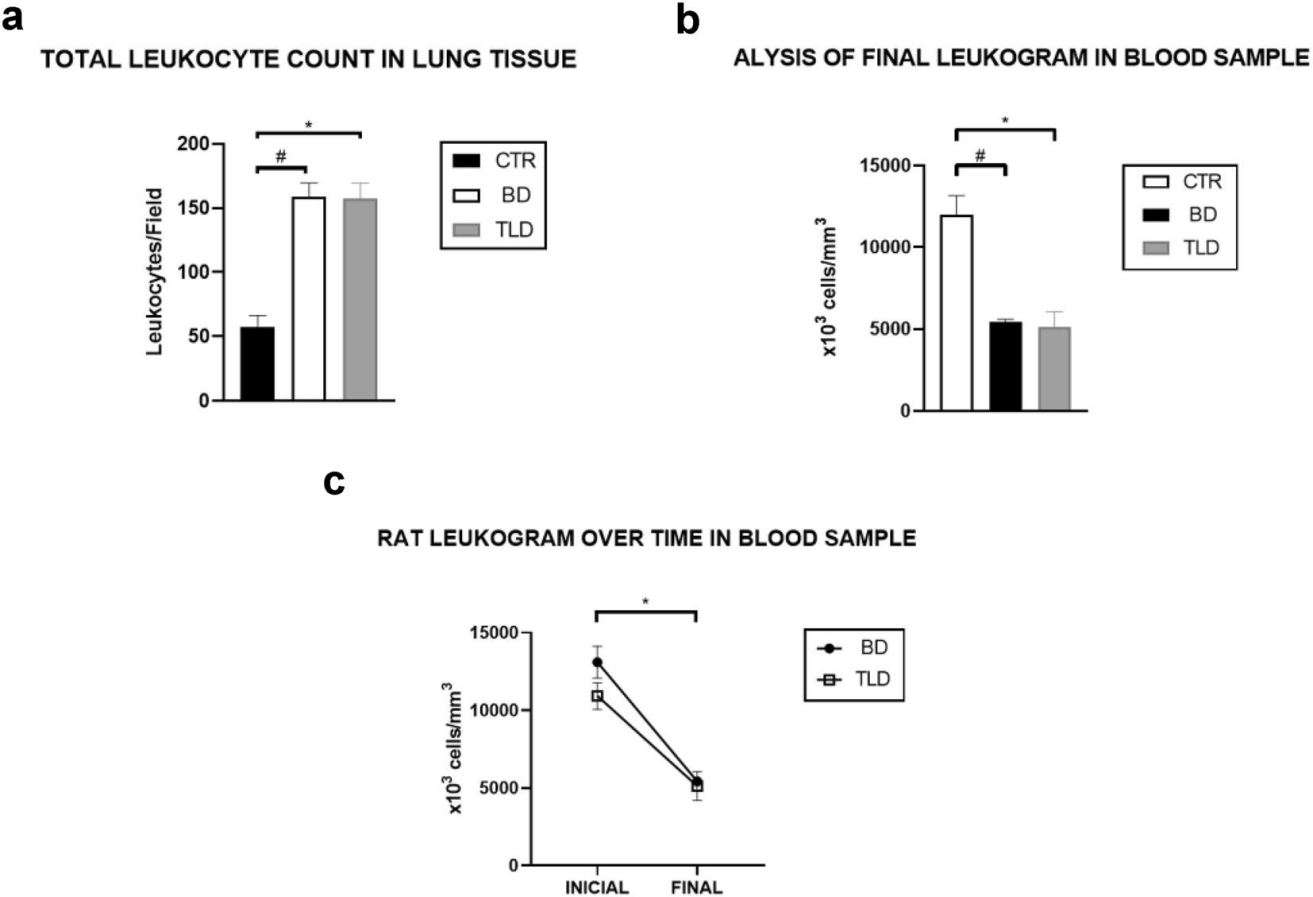
Total leukocyte count in lung tissue (**a**), analysis of the final leukocyte count in blood samples (**b**) and rat leukocyte count over time in blood samples (**c**). Values are expressed as the mean ± standard error of the mean, and groups were tested using one-way analysis of variance (ANOVA) with Tukey’s post hoc test (**a**,**b**) and two-way ANOVA with Sidak’s post hoc test (**c**). A p value < 0.05 was considered to indicate statistical significance.

In terms of the total leukocyte count in the lung tissue slides, a significant increase by 2.7 times was observed in the BD groups (BD and TLD) compared to the CTR group (Fig. 6a).

### Leukocyte count

At the end of the experiment, there was a significant decrease in the total number of leukocytes in the peripheral blood, as determined in the Neubauer chamber for both the BD group (decrease by 2.2 times) and the TLD group (decrease by 2.3 times) compared to the CTR group. However, no statistically significant differences were found between the two BD groups (Fig. 6b).

In the analysis of the Neubauer chamber leukocyte count for peripheral blood, a significant decrease in the cell count was observed over time; that is, in both the BD and TLD groups, the cell count at the final time point was significantly lower than that at the initial time point (Fig. 6c).

### Neutrophil count

**Figure 7:**
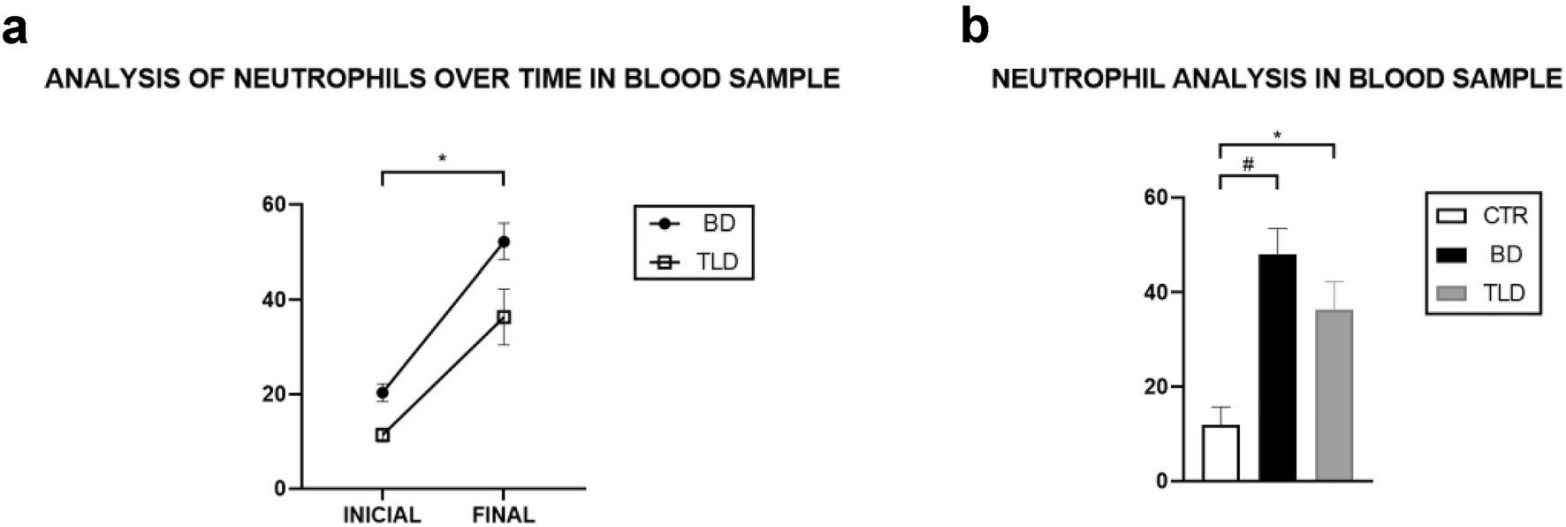
Analysis of neutrophils in blood samples over time (**a**) and neutrophil analysis in blood samples (**b**). Values are expressed as the means ± standard errors of the means, and the groups were tested using analysis of variance (two-way ANOVA) with Sidak’s post hoc test and (one-way ANOVA) with Tukey’s post hoc test. For both tests, a p value of < 0.05 was considered to indicate statistical significance.

In the analysis of neutrophils using the blood extension technique, the cell count at the final time point was significantly higher than that at the initial time point in both the BD and TLD groups (Fig. 7a). Further analysis revealed that the percentage of neutrophils in the BD group increased by 4 times more when compared to the CTR group, while in the TLD group the increase was 3 times more (Fig. 7b).

## Discussion

A lack of organ donors directly impacts the mortality of patients with end-stage respiratory disease who are waiting in line for lung transplantation. In addition, delay in performing surgery worsens the condition of the recipient, which also contributes to increased posttransplantation mortality. The utilization rates of donated lungs converted to transplantation are very low, and studies aimed at treating donors are important for improving both the quality of the organ and the performance of the graft posttransplantation. The present study evaluated a therapeutic approach based on the use of thalidomide in an experimental model of an organ donor undergoing BD^13^.

When assessing the MAP data, we found no statistically significant difference between the groups at baseline. This result was expected since randomization contributed to the homogeneity of the sample of baseline results. In our study, we observed hypotension in the first 60 min after BD induction; however, after 150 min, the blood pressure levels returned to normal. These results are consistent with those in several articles that describe arterial hypotension during BD, which occurs due to autonomic discharge followed by intense vasodilation. Studies have shown that after 1 h of BD, the blood pressure levels were comparable to those of SHAM animals (control)^14–17^.

Pulmonary oedema is frequently reported in the literature as a consequence of BD due to increased intracranial pressure and is characterized as neurogenic. Barklin et al.^18^ showed that BD resulted in an increase in the wet/dry weight ratio of the apical and basal lobes in a swine model. In our study, we also observed an increase in the wet/dry weight ratio of the lungs; however, the administration of thalidomide did not decrease the area of pulmonary oedema. These findings are different from those of Dong et al.^19^, who showed that the drug was able to significantly reduce the area of pulmonary oedema. However, these researchers used a model of injury induced by bleomycin in rat lungs^18,19^.

Previous experimental results have shown a significant increase in the levels of inflammatory mediators IL-1β, IL-6 and TNF-α up to 360 min after BD induction^6,20^. In contrast, experiments investigating BD induction models after 30–240 min found that the levels of TNF-α, IL-1β, IL-6 and IL-10 were not significantly altered^21,22^. Thus, we chose to monitor the BD induction model for 6 h.

Our group previously showed that thalidomide had beneficial effects on liver tissue samples by reducing the serum levels of liver enzymes, TNF-a, IL-1β, and IL-6; the number of macrophages and major histocompatibility complex (MHC) class I and II molecules; and the activation of NF-κB. In the kidney, we showed that thalidomide acted via a reduction in TNF-α and IL-6 levels. All of these acts to significantly inhibit the immunological response and immunogenicity of the graft^23,24^.

In our study, the level of the inflammatory marker IL-10 was not significantly different between the studied groups. In contrast, compared with that in the CTR group, the expression level of the IL-6 marker in the BD and TLD groups was significantly increased; however, although the expression level in the group treated with thalidomide tended to be lower than that in the BD group, the difference was not significant. Analysis of TNF-α revealed a significant increase in the levels in BD group compared to the CTR group and TLD group, indicating that the induction of BD generated an inflammatory process and that, according to the level of this marker, thalidomide was able to reduce this inflammation to levels approaching those of the CTR group. We also observed that the levels of IL-1β were greater in the BD and TLD groups than in the CTR group and greater in the BD group than in the TLD group, reinforcing the hypothesis that BD generates an inflammatory process and the effective action of thalidomide on this marker. These results are in agreement with findings in the literature and in previous studies by our group with samples of liver and kidney tissue^23–25^.

According to immunohistochemical analysis, BD increased the activation of NF-ĸB through the generated inflammatory process, while thalidomide significantly reduced this activation to levels equal to those of the control group. These findings are in agreement with published studies and were also demonstrated in a previous study by our group, which confirmed the anti-inflammatory effects of thalidomide on liver tissue through a significant reduction in the number of M1 macrophages^23^. Considering the mechanism of action of thalidomide, we expected an increase in IL-10 expression and a significant decrease in IL-6 expression in the TLD group. We believe that the dose or exposure time of the drug may not have been sufficient. In this sense, further studies are needed to evaluate the efficacy of the drug in this context.

Regarding pulmonary changes, Liu et al.^25^ described the protective effect of thalidomide on pulmonary lesions caused by intoxication in an experimental model. However, the effect of the drug was directly related to the dose applied, and doses of 50, 100 and 200 mg/kg were tested. Thalidomide can significantly reduce the levels of MDA, TNF-α, and IL-6 and the phosphorylation of IκB-α and p65, in addition to increasing SOD activity^25^. Our study showed similar results, as MDA levels were significantly lower in the TLD group than in the CTR group; however, SOD activity was not altered by the medication. The effects of thalidomide were also demonstrated by Dong et al.^19^, who reported that the drug notably decreased the levels of MDA and ROS and increased the level of SOD. These results suggest that the drug can significantly suppress inflammation and oxidative stress^19^.

In our study, in the evaluation of MPO, the group that received thalidomide had increased levels compared to the CTR group. Amirshahrokhi^26^ showed a reduction in MPO activity in animals treated with this drug, but the model involved inflammation induced by paraquat (an herbicide), and the medication was administered intraperitoneally over six days (50 mg/kg/ip). Due to the clinical urgency to which organ donors are subjected, our model does not have a long time to perform the treatment; thus, it was essential to work with an acute treatment protocol.

In the analysis of both pulmonary perivascular oedema and the lung injury score, no significant changes were observed between the studied groups. In contrast, Zheng^27^ showed the protective effects of thalidomide (60 mg/kg, orally) in a model of paraquat-induced lung injury in rats and cells. In that study, the drug reduced paraquat-induced oedema and the inflammatory score in the lung tissues of rats. It should be noted that the treatment adopted by the previous study was administered chronically (6 days) and involved a higher concentration than that used in our study^27^.

An increase in the total leukocyte count in the lung tissue was observed in the BD and TLD groups compared to that in the CTR group. This increase is probably related to the recruitment of inflammatory cells to the tissue due to the BD process. Another finding of our study, which reinforces the hypothesis of our investigation, is the decrease in the total leukocyte count in the peripheral blood sample of the patients in the BD group compared to that in the CTR group.

A comparison of the initial and final WBCs in peripheral blood samples revealed a significant decrease in both groups subjected to the BD process, suggesting that BD was able to promote leukopenia, a finding also confirmed by Ruiz^15^. Simas et al.^21^ demonstrated the effects of BD and trauma on the mesenteric microcirculation of rats. In this study, the number of rolling leukocytes in rats subjected to BD significantly decreased over time (50% at 30 min and 73% at 180 min); these results are in agreement with our findings^21^.

The number of neutrophils increased significantly over time in both groups of animals who underwent BD. Compared to those in the CTR group, the number of neutrophils in both the BD and TLD groups increased at the end of the study, indicating the development of neutrophilia due to the inflammation caused by the BD process. This result is in agreement with the increase in MPO observed in these groups, as the MPO enzyme is stored in neutrophil granules, and both have a strong influence on acute inflammatory processes^28^. However, our findings showed that although the drug did not result in levels matching those in the CTR group, the levels in the treatment group demonstrated a downwards trend, suggesting that the dose/exposure time of thalidomide may not have been sufficient to affect this marker.

As mentioned previously, the process of brain death leads to a pro-inflammatory state in the donor lung, which deteriorates its quality. To preserve lung quality, studies use different forms of treatment to reduce this inflammation, like we found in studies involving the use of methylprednisolone, and carbon monoxide inhalation ^29–31^.

Van Zanden et al. demonstrated that an intermediate dose of 12.5 mg/kg of methylprednisolone intravenously is the ideal treatment dose for BD-induced lung inflammation in rats, in addition to reducing the pro-inflammatory state by reducing of tissue levels of TNF-α, IL-6 and IL-1β, and promote a protective response through the expression of IL-10. Pilla et al. also showed in their study that 30 mg/kg of methylprednisolone, 5 and 60 min after induction of BD, intravenously also reduced the levels of TNF-α in lung tissue. In the study by Zhou et al. they demonstrated that carbon monoxide inhalation was able to reduce lung injury (anti-inflammatory action, due to the reduction of IL-6 and TNF- α and antiapoptotic agents) in rats subjected to brain death^29–31^.

Our study addresses a scenario in which potential organ donors are refused due to lesions caused by BD. Research aimed at treating the donor to promote adequate organ maintenance may contribute to an increase in the number of organs destined for transplantation and, consequently, to a reduction in the transplant waiting list and mortality. All inflammatory markers evaluated in this study were chosen because they are the main markers involved in the transplant/graft rejection process. There are no studies in the literature involving the action of thalidomide on the lungs of animals subjected to BD and its associations with the inflammatory process.

Injectable treatment with thalidomide via an intraperitoneal route showed important results, particularly a reduction in lung inflammation in BD donors according to the levels of markers TNF-α, IL-1β, and NF-ĸB; however, the medication was not effective for the markers IL-6 and IL-10, which may be due to the duration/dose of drug action and the acute nature of the treatment, and there are promising outcomes related to the anti-inflammatory action of the drug in the literature. Therefore, new studies are essential for understanding the action of thalidomide and for developing a BD protocol to achieve safe and effective responses in the treatment of BD donors.

We conclude that BD promotes significant acute lung inflammation, pulmonary oedema, and systemic leukopenia. According to the model and treatment duration studied, when analysing the effect of thalidomide on inflammation and oedema generated in lung tissue, the drug was found to be responsible for decreasing the levels of TNF-α, IL-1β and NF-κB, precursor compounds of the inflammatory process. In addition, thalidomide tended to decrease the level of IL-6 and the number of circulating neutrophils.

There were no significant changes in blood gas, metabolic or lung mechanics data.

## Methods

This study was approved by the Committee on Ethics in the Use of Animals (CEUA), Faculdade de Medicina, Universidade de São Paulo. The protocol was based on ethical principles and was established in accordance with the current standards of the Brazilian College of Animal Experimentation (Colégio Brasileiro de Experimentação Animal, COBEA). SDC 4512/17/012 and CEUA 946/2017. All animals were treated according to Brazilian regulations for the use of animals in scientific research and received care in accordance with international standards of animal care and experimentation in compliance with the ARRIVE guidelines.

### Study design

Twenty-four adult male *Wistar* rats weighing between 340 and 450 g were used. The animals were divided into the following experimental groups: control, n = 8 (CTR)—animals subjected to heart-lung block removal; brain death, n = 8 (BD)—animals treated with 0.9% saline solution (2 mL/kg ip) 10 min after brain death; and thalidomide, n = 8 (TLD)—animals treated with thalidomide (50 mg/kg ip) 10 min after brain death.

### Anaesthesia and surgical intervention

The animals were anaesthetized in a closed chamber with 5% isoflurane (Harvard Apparatus, Model 683, MA, USA). In the brain death group, asepsis was performed, followed by shaving the neck, cranial region and anterior femoral region. Then, the right femoral artery and vein were exposed, and the arterial catheter was connected to a pressure transducer coupled to a monitor (DIXTAL^®^, DX 2021, Brazil) to record the mean arterial pressure (MAP). The femoral vein catheter was intended for the infusion of 0.9% saline solution (hydration), and blood aliquots were removed for blood gas analysis at the end of the experiment (360 min).

### Induction of brain death

BD was induced by drilling into the subdural region of the skull and inserting a Fogarty-4F catheter (Edwards Life Science LLC, Irvine, CA, USA), which was rapidly insufflated with 0.5 mL of saline solution. BD was confirmed by bilateral mydriasis, apnoea, absence of reflexes, a hypertensive peak and a decrease in MAP.

### Evaluation of the wet weight/dry weight ratio

The wet weight was measured on a semianalytical scale. Then, the caudal lobe was subsequently dried in an oven at 80 °C for 72 h. At the end of drying, the dry weight of the lung was assessed. The wet/dry weight ratio was calculated by dividing the wet by the dry weight.

### Evaluation of inflammatory mediators and oxidative stress

At the end of the experiment, lung tissue samples were collected in Eppendorf tubes and stored at − 80 °C. The levels of the following inflammatory mediators in the homogenized lung tissue were measured using commercially available enzyme-linked immunosorbent assays (ELISAs): TNF-α (RAB0480; COMPANY SIGMA-ALDRICH), IL-6 (RAB0312; COMPANY SIGMA-ALDRICH), IL-1β (RAB0278; COMPANY SIGMA-ALDRICH) and IL-10 (E0108Ra; COMPANY BT LAB). The levels of reactive oxygen species (ROS) were evaluated via the nitrotrazolium blue salt assay (Sigma‒Aldrich, St. Louis, USA)^32^. Superoxide dismutase (SOD) activity was measured by monitoring the inhibition of epinephrine autooxidation (Sigma‒Aldrich, St. Louis, USA)^33^. Catalase activity (CAT) was measured by the rate of decrease in hydrogen peroxide concentration (Sigma‒Aldrich, St. Louis, USA)^34^. As an index of oxidative damage induced by lipid peroxidation, we used the thiobarbituric acid-reactive substances (TBARS) method (Sigma‒Aldrich, St. Louis, USA) to analyse malondialdehyde (MDA)^35^. Myeloperoxidase (MPO) activity was measured using hydrogen peroxide, hexadecyltrimethylammonium bromide (HTAB; Sigma‒Aldrich, St. Louis, USA) and tetramethylbenzidine (TMB; Sigma‒Aldrich, St. Louis, USA)^36^. The kits and analyses were performed according to the manufacturer’s instructions.

### Immunohistochemical analysis of lung tissue

Immunohistochemical analysis was performed by staining white slides with an anti-NF-κB antibody (p65) (Sigma‒Aldrich). The entire tissue section was scanned under a light microscope, and ten random fields were chosen at 40x magnification. The brown-stained area around the blue-stained cell nucleus was defined as the region of interest.

### Pulmonary histopathological evaluation

The cranial lobe of the right lung was collected, fixed and stained on a slide with haematoxylin and eosin. The slides were scanned for subsequent analysis of perivascular oedema and calculation of the lung injury score using ImageJ image analysis software.

### WBC analysis

To count the total number of leukocytes, 20 µL samples of peripheral blood were collected from the animals’ tails and added to 380 µL of Turk’s solution to quantify the total number of leukocytes in a Neubauer chamber.

For the differential determination of leukocytes, the blood extension technique was used to stain cells via the Instant Prov^®^ method (Newprov PR, Brazil).

### Statistical analyses

Results are expressed as the mean ± standard error of the mean (SEM). The graphs were produced and statistically analysed using Graph Pad Prism 8 software. The results were considered significant when the probability of occurrence of the null hypothesis was lower than 5% (p < 0.05). The analyses of normality and equality of variance were performed using the Shapiro‒Wilk test and Levene test, respectively. The data from two groups were compared using Student’s t test. To compare single factors among more than two groups, one-way ANOVA with Tukey’s post hoc test was used, while two-way ANOVA with Sidak’s post hoc test was used to compare two factors among more than two groups.

## Data Availability

All relevant data supporting the findings of this study are already available in the manuscript, however, upon reasonable request, raw data will be made available by the corresponding author.
